# Role of Cleaved PINK1 in Neuronal Development, Synaptogenesis, and Plasticity: Implications for Parkinson’s Disease

**DOI:** 10.3389/fnins.2021.769331

**Published:** 2021-11-02

**Authors:** Smijin K. Soman, Ruben K. Dagda

**Affiliations:** Department of Pharmacology, School of Medicine, University of Nevada, Reno, Reno, NV, United States

**Keywords:** cleaved PINK1, Parkinson’s disease, PKA signaling, BDNF (brain derived neurotrophic factor), mitochondrial retrograde signaling, neuronal plasticity and neurogenesis

## Abstract

Mitochondrial dysfunction plays a significant role in the pathogenesis of Parkinson’s disease (PD). Consistent with this concept, loss of function mutations in the serine/threonine kinase- PINK1 (PTEN-induced putative kinase-1) causes autosomal recessive early onset PD. While the functional role of f-PINK1 (full-length PINK1) in clearing dysfunctional mitochondria via mitophagy is extensively documented, our understanding of specific physiological roles that the non-mitochondrial pool of PINK1 imparts in neurons is more limited. PINK1 is proteolytically processed in the intermembrane space and matrix of the mitochondria into functional cleaved products (c-PINK1) that are exported to the cytosol. While it is clear that posttranslational processing of PINK1 depends on the mitochondria’s oxidative state and structural integrity, the functional roles of c-PINK1 in modulating neuronal functions are poorly understood. Here, we review the diverse roles played by c-PINK1 in modulating various neuronal functions. Specifically, we describe the non-canonical functional roles of PINK1, including but not limited to: governing mitochondrial movement, neuronal development, neuronal survival, and neurogenesis. We have published that c-PINK1 stimulates neuronal plasticity and differentiation via the PINK1-PKA-BDNF signaling cascade. In addition, we provide insight into how mitochondrial membrane potential-dependent processing of PINK1 confers conditional retrograde signaling functions to PINK1. Further studies delineating the role of c-PINK1 in neurons would increase our understanding regarding the role played by PINK1 in PD pathogenesis.

## Introduction

Parkinson’s disease (PD) is the fastest-growing neurodegenerative disorder, predominantly characterized by extensive and progressive loss of dopaminergic neurons in the *substantia nigra pars compacta* (SNpc) of the midbrain (Dorsey et al., 2018). Specifically, PD is characterized by classical motor symptoms including rigidity, tremor, and bradykinesia that manifest in one or two limbs in mid-stage PD and progressing to upper and lower extremities with the presence of gait imbalance in advanced forms of PD; however, beyond motor symptoms, the progression of PD is often associated with non-motor symptoms such as dementia and major clinical depression (Massano and Bhatia, 2012; Biundo et al., 2016; Piredda et al., 2020). While the pathogenesis of PD remains elusive, mounting evidence suggests a convergence of mitochondrial and proteasomal dysfunction as major etiological factors that contribute to PD pathogenesis. The contribution of mitochondrial dysfunction to PD pathology is discernible, as evidenced by data gained from an array of experimental and genome-wide association studies (International Parkinson Disease Genomics Consortium et al., 2011) showing mitochondrial injury (ROS-mediated damage of complex proteins, loss of transmembrane potential, halting of the TCA, and Ox/Phos cycles) in dopamine neurons as a causative factor of PD pathology (International Parkinson Disease Genomics Consortium et al., 2011; Malpartida et al., 2021). Also, studies performed in postmortem PD patient brain tissue have shown decreased mitochondrial biomass, bioenergetic capacity, and altered mitochondrial distribution of mitochondria within the cell bodies and neurites (Schapira et al., 1990; Keeney et al., 2006; Navarro et al., 2009; Mallach et al., 2019). Thus, therapies that reverse mitochondrial dysfunction are a promising therapeutic approach; however, various human clinical trials targeting mitochondrial dysfunction in PD have been unsuccessful in reversing the course of disease (e.g., disease progression and motor symptoms) (Beal et al., 2014; Kieburtz et al., 2015; Prasuhn et al., 2021). Thereby, additional basic science studies that functionally dissect the role of the various mitochondrial localized PD-linked gene products and their function are essential to broaden our understanding of mitochondria and their role in PD pathogenesis, ultimately leading to targeted therapies against PD.

Over more than 50 loss of function mutations in PTEN−induced kinase 1 (PINK1) leading to PD contributes to the second most common form of autosomal recessive early onset PD (Valente et al., 2004a; Kawajiri et al., 2011). PINK1 is a large, atypical serine/threonine kinase, localized in both cytosolic and mitochondrial compartments, with a preponderant role in eliminating aged and dysfunctional mitochondria through a well-orchestrated physiological process termed mitophagy (autophagosome/lysosome mediated mitochondrial turnover) (Narendra et al., 2010). As characterized in a myriad of primary research papers, f-PINK1 (full-length PINK1) predominantly mediates mitophagy in non-neuronal cells, as well as neurons. However, beyond f-PINK1-mediated mitophagy and adding a layer of functional complexity to its multi-faceted roles in neurons, emerging evidence shows that PINK1 is a multifunctional ser/thr kinase that is proteolytically processed by mitochondria into c-PINK1 (cleaved PINK1) products with lower molecular weight isoforms (52, 44, 36 kDa). These cleaved products are extruded to the cytosol and diffusely localized in various neuronal sub-compartments such as dendrites, axons and soma to modulate different neuronal functions. However, the emphasis on f-PINK1 and its role in mitophagy has significantly curtailed the field’s attention and resources necessary to understand the extra-mitochondrial role of PINK1 in cellular function. Herein, we review the non-canonical roles of PINK1, with particular emphasis on its cleaved forms, which are reported in the literature and implicate the functional roles of c-PINK1 in neuronal function and PD pathology.

## Canonical Role of PINK1 in Mitophagy

**FIGURE 1:**
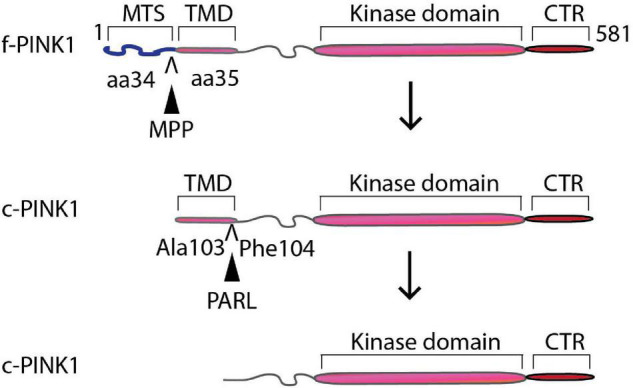
Proteolytic processing of PINK1. PINK1 gene encodes a 581 amino acid protein with an N-terminal mitochondrial targeting motif that contains a transmembrane domain (110 amino acids long), an un-conserved region, a kinase domain with three insertions in the N lobe, and a conserved C-terminal region (CTR). IMM-localized mitochondrial processing peptidases (MPP) putatively cleave PINK1 at aa 34 and 35 to form a 60 kDa intermediate cleavage product. PARL and/or m-AAA cleave the intermediate 60 kDa intermediate product in between Ala103 and Phe104 to generate 52/48 kDa processed forms of PINK1.

PINK1 gene encodes a 581 amino acid protein with an N-terminal mitochondrial targeting motif that contains a transmembrane domain (110 amino acids long), an un-conserved region, a kinase domain with three insertions in the N lobe, and a conserved C-terminal region (CTR) of unknown function and structure (Schubert et al., 2017). Mitochondrial function depends on an optimum mitochondrial membrane potential (ΔΨm) and proton gradient (ΔpH). Given its ability to bind to TOM20, intermembrane-space anchored f-PINK1 acts as a quality control checker of mitochondrial health, including mitochondrial import and proper ΔΨm (Narendra et al., 2010). However, upon loss of optimum ΔΨm, due to oxidative stress from ROS derived from the reverse flow of electrons through complexes I and II (or from complex III to IV) and via inner membrane ion leaks, the rapid translocation of the N-terminus domain of f-PINK1 from the OMM to the IMM is interrupted (Figure 1), leading to accumulation, dimerization, autophosphorylation, and subsequent activation of f-PINK1 on the OMM (Dagda et al., 2009). The activation of f-PINK1 leads to phosphorylation of Ser65 residues of ubiquitin and of ser/thr residues localized within the ubiquitin-like (UBL) domain of Parkin, thereby inducing conformational changes that allow for binding of the charged E2 ligase and enabling its E3 ubiquitin ligase activity, resulting in downstream ubiquitylation of multiple mitochondrial substrates to promote localized, targeted degradation of OMM proteins through the ubiquitin-proteasome pathway (Mallach et al., 2019; Malpartida et al., 2021). Parkin is an E-3 ubiquitin ligase that ubiquitylates certain mitochondrial substrates (MFN, Miro, Tom70A, VDAC, FIS1, HK1, ATP5A) at lysine-48 or lysine-63 (Sarraf et al., 2013). The ubiquitylated proteins on the OMM then trigger the binding of autophagic adaptors NDP52 and optineurin to anchor the OMM to the autophagosomes via LC3 to target the whole organelle for lysosomal-mediated degradation, which subsequently stimulates mitochondrial biogenesis (Heo et al., 2015; Lazarou et al., 2015; Ivankovic et al., 2016).

## Conditional Proteolytic Processing of PINK1

**FIGURE 2:**
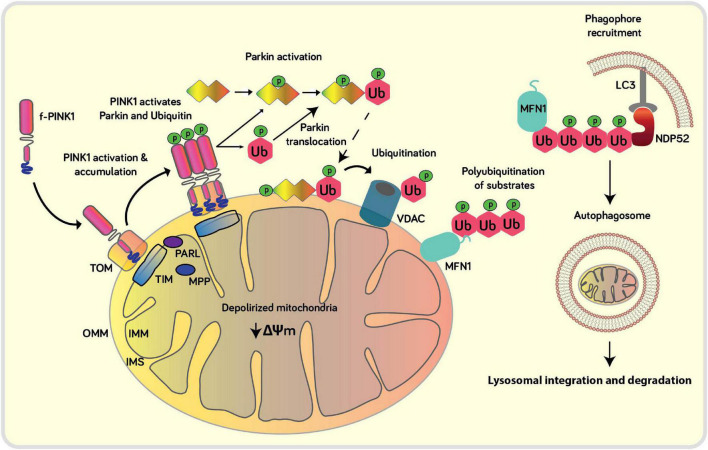
Schematic of the canonical role of PINK1 in mitophagy: Upon loss of ΔΨm the import of PINK1 into the IMM is blocked which results in the overt accumulation of PINK1 at the OMM, which blocks mitochondrial import of mitochondrially-localized proteins mediated by the TOM complex. At the OMM, PINK1 can autophosphorylate itself, phosphorylate ubiquitin and Parkin in order to recruit and activate Parkin at the damaged mitochondria. Parkin ubiquitinates several mitochondrial substrates such as Mfn, VDAC1 and Drp1. Poly-Ub chains are subsequently phosphorylated by PINK1 serving as a signal for the autophagic machinery to trigger the initiation of mitophagy. Adaptor proteins (OPTN, NDP52) recognize phosphorylated poly-Ub chains on mitochondrial proteins and initiate autophagosome formation through binding with LC3.

Conversely, under optimum ΔΨm, a low NADH/NAD^+^ ratio and coupled mitochondria, the N-terminal region of PINK1 translocates across the OMM to the inner IMM via the TOM-TIM complex, with the kinase domain located closer to the C-terminus protruding out into the cytosol. PINK1 is cleaved (Figure 2) by up to 4 different IMM-localized mitochondrial processing peptidases (MPP) to form a 60 kDa intermediate cleavage product (Greene et al., 2012; Liu et al., 2017). Cleavage by rhomboid protease Presenilin-Associated Rhomboid-like protein (PARL) and/or matrix-AAA (m-AAA) proteases produces 52/48 kDa processed forms of PINK1 (Lin and Kang, 2008; Whitworth et al., 2008; Meissner et al., 2011; Greene et al., 2012), which is retro-translocated to the cytosol for extra-mitochondrial duties or degradation via the N-end rule pathway (Yamano and Youle, 2013; Sekine and Youle, 2018). It is worth noting that a significant fraction of the endogenous pool of PINK1 is localized in the cytosolic compartment, in addition to localization in the mitochondria and microsomal fraction as assessed by densitometric analysis of PINK1 levels and mitochondrial/cytosolic markers in Western blots of subcellular fractions in neuronal cells (Dagda et al., 2009, 2014). The previous observation underscores the need to fully unveil the functional roles of extra-mitochondrial forms of PINK1 in neurons and its implication in PD and neuronal functions. Thereby, conditional processing of PINK1 into cleaved functional protein products based on optimal ΔΨm acts as a form of mitochondria-directed retrograde cell signaling.

## Exiting the Mitochondrion: PINK1 Exerts Non-Canonical Physiological Roles in the Cytosol Through Retrotranslocation

**FIGURE 3:**
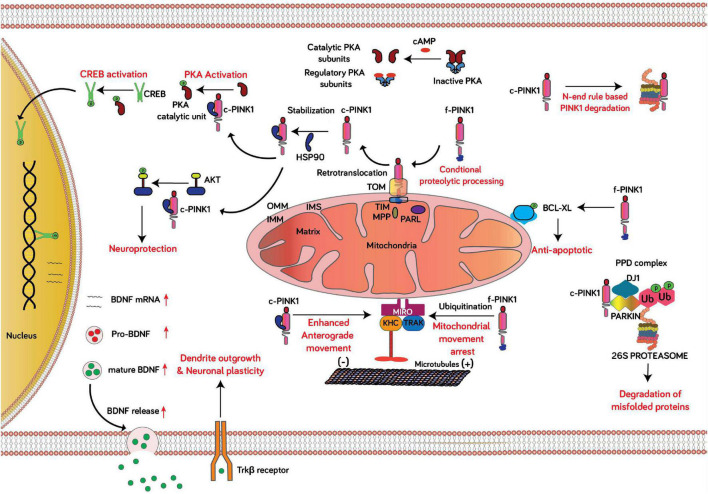
Schematic of the non-canonical role of c-PINK1 in neuronal function. Conditional proteolytic processing: In healthy neurons, most mitochondria are highly functional, as evident by an adequate ΔΨm that enables the import of PINK1 via the TOM-TIM protein import complex at the OMM and IMM, respectively. In healthy mitochondria, PINK1 is cleaved by PARL and MPP at the IMM, and the cleaved PINK1 (c-PINK1) is retro-translocated to the cytosol to exert extra-mitochondrial functions. PINK1-PKA-CREB-BDNF signaling; Mitochondria with optimum ΔΨm proteolytically processes f-PINK1 to c-PINK1. The c-PINK1 stabilizes and retro-translocated to the cytosol and phosphorylates the catalytic subunit of PKA and enhances PKA mediated CREB activation, resulting in upregulated BDNF expression and release. BDNF, a neurotrophic factor augments neuronal plasticity. c-PINK1 degradation through N-end rule pathway: The N-end rule specific E3 enzymes UBR1, UBR2, and UBR4 recognize the N-terminal phenylalanine residue of c- PINK1 for proteasomal degradation. AKT activation: Activation of PINK1 leads to phosphorylation of cytosol localized AKT. Mitochondrial movement: f-PINK1 phosphorylates Miro1 to arrest mitochondrial movement. However, c-PINK1 can activate PKA-mediated phosphorylation of Miro2 to enhance the anterograde movement of mitochondria in dendrites and axons. Anti-apoptotic: PINK1 interacts with and phosphorylates Bcl-xL, an anti-apoptotic protein, hindering the pro-apoptotic cleavage of Bcl-xL. Degradation of misfolded proteins: Parkin, PINK1, and DJ-1 form a PPD complex to promote ubiquitination and degradation of Parkin substrates and PINK1 mutation leads to decreased degradation and accumulation of abnormally folded Parkin substrates.

The relevance of PINK1 in cellular function gained widespread attention after discovering autosomal recessive mutations in PINK1 by Valente et al. (2004b) were associated with PD. Identifying the N-terminal mitochondrial targeting sequence in PINK1 suggested an exclusive localization to mitochondria (Valente et al., 2004a). However, upon performing biochemical and subcellular fractionation assays, other research groups had observed truncated versions of PINK1 (∼53 kDa) in the cytosolic fractions (Weihofen et al., 2008). Biochemical fractionation and imaging studies later confirmed that f-PINK1 is primarily localized to mitochondria, and cleaved forms of PINK1 are diffusely localized across the cytosol (Lin and Kang, 2008; Takatori et al., 2008; Weihofen et al., 2008; Xiong et al., 2009). Distinctly, mitochondria possessing optimal ΔΨm import f-PINK1 (63 kDa) and subject it to cleavage between aa 34 and 35 by MPP and then at Ala103 and Phe104 through PARL to form the 52 kDa protein isoform (c-PINK1) with intact functional property (Lin and Kang, 2010; Deas et al., 2011; Meissner et al., 2011). The c-PINK1 is retro-translocated to the cytosol and stabilized or degraded through the N-end rule pathway via the actions of the ubiquitin-protein ligase E3 component n-recognin (UBR) family of E3 ligases (Lin and Kang, 2008; Yamano and Youle, 2013). The stabilization of c-PINK1 in the cytosol is mediated via Lys-63- linked ubiquitination, and Nf-KB activation promotes the stability of cleaved PINK1 (Lim et al., 2015). Proteasomal inhibition in HeLa cells with MG132 causes accumulation of cleaved PINK1 leading to reduced Parkin translocation to mitochondria with dissipated ΔΨm (Fedorowicz et al., 2014). However, another study failed to observe translocation of Parkin in a similar experimental set-up, instead observed promotion of mitophagy through cleaved PINK1 in mitochondria not overly depolarized (Lim et al., 2015). The ability of cytosolic localized c-PINK1 to initiate mitophagy is not clear, as a few conflicting reports cite the ability or inability to phosphorylate and translocate Parkin to execute mitophagy. The discrepancies between studies reporting on the ability of cleaved PINK1 to facilitate or block mitophagy can be explained by differences in the experimental models to initiate mitophagy (e.g., treating cells with CCCP vs. valinomycin) or cell lines used. In addition, Dagda et al. (2009) showed that transient expression of cleaved, cytosolic localized PINK1 (ΔN-PINK1) can mitigate macroautophagy induced by loss of PINK1 in human, undifferentiated SH-SY5Y neuroblastoma cells (Dagda et al., 2009; Fedorowicz et al., 2014; Lim et al., 2015). Consistent with the inability of c-PINK1 to initiate mitophagy, other research reports have shown that pharmacological activation of PINK1 by treating primary cortical neurons with the ATP analog kinetin or transient expression of ΔN-PINK1 does not enhance macroautophagy under physiological conditions (Das Banerjee et al., 2017; Soman et al., 2021). It is not clear whether c-PINK1 can affect macroautophagy/mitophagy responses, especially given its ability to activate downstream PKA signaling in the cytosol, known to downregulate global autophagy via PKA-mediated phosphorylation of autophagic adaptor proteins and components (Cherra et al., 2010; Akabane et al., 2016). While f-PINK1 expression alone can suppress toxin-induced macroautophagy in SH-SY5Y cells, there is precedence for a role of c-PINK1 in suppressing macroautophagy/mitophagy induced by oxidative stress given that transient expression ΔN-PINK1 is sufficient to suppress macroautophagy induced by loss of endogenous PINK1 in neuroblastoma cells (Dagda et al., 2009). Additionally, while enhanced PKA activity (in the cytosol or mitochondria) impairs Parkin-mediated mitophagy imparted by f-PINK1, it remains to be elucidated whether enhanced downstream activation of PKA elicited by c-PINK1 directly inhibits the f-PINK1-Parkin pathway via PKA-mediated phosphorylation of MIC60 (Akabane et al., 2016). It is worth noting that the orientation of OMM bound f-PINK1 toward the cytosol and localization of c-PINK1 in the cytosol suggest the functional realm of PINK1 influence expanding from mitochondria to the cytosol (Figure 3). Evidently, the expression of c-PINK1 but not f-PINK1 is sufficient to protect dopaminergic neurons from the toxic effect of MPTP (Haque et al., 2008), and can suppress macroautophagy and neuronal death induced by loss of endogenous PINK1 in SH-SY5Y cells (Dagda et al., 2009). In another study, PINK1 enhanced the phosphorylation level of cytosolic localized AKT via activation of mTORC2 (Murata et al., 2011). AKT is crucial for dopaminergic neuronal viability, and PI3K/Akt signaling is curtailed in MPTP-induced PD rodent models (Hu et al., 2018). Furthermore, emerging experimental evidence shows that c-PINK1, which can be localized to the cytosol and is weakly anchored to the OMM, can enhance anterograde-mediated trafficking of mitochondria in neurites (both in dendrites and axons) by stimulating downstream PKA-mediated phosphorylation of the mitochondrial adaptor protein Miro2 (Rhot2 in humans) (Matenia et al., 2012; Dagda et al., 2014). However, f-PINK1 has been shown to stimulate mitophagy of synaptic mitochondria by enhancing the degradation of the mitochondrial adaptor protein Miro-1 (Rhot1) to stall mitochondria and ensure their subsequent autophagic-lysosomal clearance (Wang et al., 2011); however, it is not clear to date whether c-PINK1, or f-PINK1 for that matter, can stimulate mitophagy of oxidatively-damaged mitochondria localized within dendritic trees of neurons.

The delineation of PINK1 as a functional cytosolic kinase was fortified with the discovery that cytosolic c-PINK1 promotes neuronal plasticity and differentiation. PINK1-deficient cortical and midbrain neurons show signs of reduced dendritic trees and can be rescued by introducing c-PINK1 (ΔN-PINK1) but not OMM localized PINK1 (Dagda et al., 2014). Moreover, transient over-expression of c-PINK1 induces neuronal differentiation in SH-SY5Y human dopaminergic neuroblastoma cells, as evident from increased levels of differentiation markers through activation of Protein kinase A (PKA) signaling (Dagda et al., 2014). Later studies confirmed that f-PINK1 binds and phosphorylates the catalytic subunit of PKA at T197 (PKAcat, pT197), resulting in activation of PKA downstream signaling (Wang et al., 2018). Overall, given that PKA is a well-characterized ser/thr kinase critical for maintaining essential neuronal functions—including mitochondrial homeostasis, bioenergetics, neuronal development, and neurotransmission-, c-PINK1 acts as a regulator of neuronal development and differentiation by acting as an upstream gatekeeper of PKA activity in the cytosol by enhancing the autocatalytic activation of the catalytic subunit of the PKA holoenzyme. In the context of oxidative stress and in PD, the PINK1 knockout mouse exhibits loss of neuronal development markers such as GFAP, a phenotype that is associated with significantly reduced global PKA activity in the brain, cellular pathology that coincides with significantly reduced mitochondrial content and dendritic arbors in midbrain dopamine neurons (Das Banerjee et al., 2017). Although several extra-mitochondrial roles of PINK1 have been reported in literature, it is worth noting that some of these newfound physiological roles of PINK1 have not been linked to c-PINK1 while other roles have. For instance, in addition to regulating the differentiation of neurons, it has been observed that the endogenous levels of PINK1 are elevated during embryonic and postnatal brain development to regulate the differentiation of astrocytes (Choi et al., 2016). Beyond the brain, it is worth noting that PINK1 protein expression can also be upregulated in breast, colorectal, and endometrial cancer tissues, whereas PINK1 inhibition restricts cancer cell proliferation (Zhang et al., 2017). Furthermore, PINK1 has been observed to influence the differentiation of hippocampal neurons as PINK1-KO mice exhibit impaired metabolic capacity leading to abrogated differentiation of hippocampal neuronal stem cells (Agnihotri et al., 2017). Additionally, recent evidence from EdU analyses and lineage-tracing studies in zebrafish and human organoid models suggest that PINK1 deficiency impedes dopaminergic neuron neurogenesis during adulthood (Brown et al., 2021). However, the observations that PINK1 can regulate cytosolic-localized signaling pathways in neurons to modulate stem cell differentiation and hippocampal differentiation suggest an involvement of c-PINK1; however, it is clear that futures studies are warranted to determine if any cleaved products of PINK1 regulate/facilitate these physiological functions or rule out f-PINK1 as well. In aggregate, present evidence points out that PINK1 acts as a crucial regulator of neuronal development, presumably by activating downstream PKA. A recent report unraveled the possible mechanism through which PINK1 influences neuronal development by modulating the PKA-mediated production and subsequent extracellular release of brain-derived neurotrophic factor (BDNF). Specifically, pharmacological activation of PINK1 by treating cortical neurons with kinetin (pharmacological activator of PINK1) enhances the downstream-mediated autocatalytic activation of PKA. Phosphorylated PKA then phosphorylates the transcription factor CREB in the cytosol, which subsequently translocates to the nucleus to enhance the production of mature, cleaved BDNF. The increased level of BDNF is associated with increased activation and phosphorylation of the BDNF receptor Tropomyosin Receptor Kinase B which thereby enhances BDNF-mediated neuronal differentiation, as evident by an increase in the levels of presynaptic and postsynaptic markers in neurons (Soman et al., 2021). For several decades, neuroscientists have widely accepted that neuronal development and plasticity, especially in the hippocampus and cerebral cortex, is predominantly mediated by the calcium-calcium calmodulin and CAM Kinase pathways, which affect long-term memory potentiation and memory consolidation. The recent papers documenting a possible role of PINK1 in mediating synaptogenesis and neuronal development require further investigation to determine the extent to c-PINK1 and PKA interplay with the canonical CAM Kinase/calcium calmodulin signaling pathways in neuronal development.

## Conclusion

While additional studies are warranted to further functionally dissect the newfound c-PINK1-PKA-CREB-BDNF signaling axis and its role in mediating neuronal survival, differentiation, and interplay with the f-PINK1-Parkin mitophagy, it is clear that c-PINK1 has an underappreciated role in neuronal development, plasticity which is distinct from its canonical role in regulating mitochondrial quality control, structure, and function. In conclusion, this mini-review sheds new “light” on the possible role of cytosolic-localized c-PINK1 variants as critical molecular players in regulating neuronal functions, including the development of neuronal circuits, modulation of neuronal survival, enhancing the arborization of dendrites in developing neurons, and the formation and maturation of dendritic spines, which deserves attention beyond what is known about mitochondrial quality control regulation by mitochondrial-localized PINK1.

## Author Contributions

SS and RD researched literature, conceived the presented concepts, and wrote the manuscript. Both authors contributed to the manuscript and approved the submitted version.

## Conflict of Interest

The authors declare that the research was conducted in the absence of any commercial or financial relationships that could be construed as a potential conflict of interest.

## Publisher’s Note

All claims expressed in this article are solely those of the authors and do not necessarily represent those of their affiliated organizations, or those of the publisher, the editors and the reviewers. Any product that may be evaluated in this article, or claim that may be made by its manufacturer, is not guaranteed or endorsed by the publisher.
